# An effective prognostic model in colon adenocarcinoma composed of cuproptosis-related epigenetic regulators

**DOI:** 10.3389/fphar.2023.1254918

**Published:** 2023-08-24

**Authors:** Yang Liu, Yizhao Wang, Chang Li, Huijin Feng, Yanqing Liu, Lianjun Ma

**Affiliations:** ^1^ Endoscopy Center, China-Japan Union Hospital of Jilin University, Changchun, China; ^2^ Department of VIP Unit, China-Japan Union Hospital of Jilin University, Changchun, China; ^3^ School of Life Sciences, Nanjing University, Nanjing, China

**Keywords:** colon adenocarcinoma (COAD), cuproptosis, epigenetic regulator, mRNA expression, prognostic model

## Abstract

**Background:** Colorectal adenocarcinoma (COAD) is a common malignant tumor with little effective prognostic markers. Cuproptosis is a newly discovered mode of cell death that may be related to epigenetic regulators. This study aimed to explore the association between epigenetic regulators and cuproptosis, and to establish a prognostic prediction model for COAD based on epigenetic regulators associated with cuproptosis (EACs).

**Methods:** RNA sequencing data and clinical data of 524 COAD patients were obtained from the TCGA-COAD database, cuproptosis-related genes were from the FerrDb database, and epigenetic-related genes were from databases such as GO and EpiFactors. LASSO regression analysis and other methods were used to screen out epigenetic regulators associated with cuproptosis and prognosis. The risk score of each patient was calculated and the patients were divided into high-risk group and low-risk group. Next, the survival difference, functional enrichment analyses, tumor mutation burden, chemotherapy drug sensitivity and other indicators between the two groups were compared and analyzed.

**Results:** We found 716 epigenetic regulators closely related to cuproptosis, among which 35 genes were related to prognosis of COAD. We further screened out 7 EACs from the 35 EACs to construct a prognostic prediction model. We calculated the risk score of each patient based on these 7 genes, and divided the patients into high-risk group and low-risk group. We found that the overall survival rate and progression-free survival rate of the high-risk group were significantly lower than those of the low-risk group. This model showed good predictive ability in the training set, test set and overall data set. We also constructed a prognostic prediction model based on risk score and other clinical features, and drew the corresponding Nomogram. In addition, we found significant differences between the high-risk group and the low-risk group in tumor mutation burden, chemotherapy drug sensitivity and other clinical aspects.

**Conclusion:** We established an effective predictive prediction model for COAD based on EACs, revealing the association between epigenetic regulators and cuproptosis in COAD. We hope that this model can not only facilitate the treatment decision of COAD patients, but also promote the research progress in the field of cuproptosis.

## Introduction

Colon adenocarcinoma (COAD) is a malignant tumor that originates from the colon (Siegel et al., 2023a). It is the major type of colorectal cancer. Variable stages of COADs appear to have quite distinct 5-year relative survival rates, with 90% for localized disease, 72% for regional disease, and only 14% for distant metastatic COAD (Siegel et al., 2023b). Despite extensive researches, definitive prognostic factors for COAD have not been established yet. Age at diagnosis, stage, and genomic biomarkers such as p53 mutations and microsatellite instability have been identified as potential prognostic factors, but more reliable biomarkers are needed to aid clinical assessment of COAD.

Cuproptosis is a new form of cell death that is triggered by intracellular copper accumulation (Tsvetkov et al., 2022). It is different from formerly identified other types of cell deaths, such as apoptosis, ferroptosis, and necroptosis. Cuproptosis involves a mitochondrial proteotoxic stress that causes lipoylated proteins to aggregate and iron-sulfur cluster proteins in the mitochondria to become unstable (Tsvetkov et al., 2022). Energy depletion and cell death arise from these events, which disrupt the tricarboxylic acid (TCA) cycle and the mitochondrial electron transport chain. Cuproptosis may have therapeutic significance for cancer treatment because copper homeostasis and metabolism are frequently disrupted in cancer cells (Shanbhag et al., 2021). Copper levels in cancer cells are typically higher than in normal cells, which may give them an advantage during angiogenesis, invasion, and metastasis, but also result in enhanced vulnerability to cuproptosis (Xie et al., 2023). Therefore, cuproptosis could serve as a viable therapeutic target in the fight against cancer, including COAD (Cao et al., 2023).

Epigenetics is about how environmental factors and behavioral modifications can modify our gene function without altering the DNA sequence (Cavalli and Heard, 2019). By “turning on” or “turning off” genes through a variety of methods, including DNA methylation, histone modification, and non-coding RNA, epigenetic regulators can affect how genes are expressed, which has significant implications for human health and disease (Sharma et al., 2010; Cavalli and Heard, 2019; Gao et al., 2020). Studies have emphasized that epigenetic changes play crucial roles in the initiation and development of COAD (Lao and Grady, 2011; Liu et al., 2017; Liu et al., 2018). Epigenetic alterations also influence the patient’s prognosis and response to treatment (Koch et al., 2018).

Copper has been shown to regulate epigenetic pathways (Michniewicz et al., 2021). Thus, accompanying the cuproptosis, there may be some epigenetic changes. Moreover, by altering the expression and activity of cuproptosis-related genes (CRGs), epigenetic regulators can modify how sensitive the cells are to cuproptosis. For instance, p53 controls metabolism, DNA repair, apoptosis, and cell cycle (Liu et al., 2019; Liu and Gu, 2022a; Liu and Gu, 2022b). Particularly, p53 can limit glycolysis and promote TCA cycle and oxidative phosphorylation in cancer cells, increasing their propensity for cuproptosis. Additionally, p53 can control the biogenesis of iron-sulfur clusters and the production of glutathione, a copper chelator that contains thiols and shields cells against cuproptosis (Liu and Gu, 2022a). Therefore, p53 is proposed to be a potential master regulator of cuproptosis (Xiong et al., 2023). The expression and activity of p53 are tightly regulated by many epigenetic regulators and mechanisms (Saldana-Meyer and Recillas-Targa, 2011). It is reasonable to speculate that epigenetic regulators can affect cuproptosis via p53. The expression of CRGs such as FDX1 (a ferredoxin implicated in cuproptosis) and SLC31A1 (a copper importer) can be regulated by epigenetic changes such as DNA methylation and histone modifications (Wu et al., 2021; Xu et al., 2022). These epigenetic alterations can modify a cell’s susceptibility to cuproptosis by affecting the mitochondrial activity and intracellular copper levels. Taken together, cuproptosis may be intertwined with epigenetics in multiple ways. How their link is relevant to the pathology and treatment of COAD is not clear. However, based on their respective significance in COAD, exploring this link may generate exciting results regarding to the prognostic and therapy of COAD.

In this study, we analyze the CRGs in COAD samples and identified an effective prognostic signature made up of 7 cuproptosis-related epigenetic regulators. This model does not only efficiently predict the survival of COAD patients, but also give us much interesting information about the pathological relevance of cuproptosis in COAD.

## Materials and methods

### Data acquisition

**FIGURE 1:**
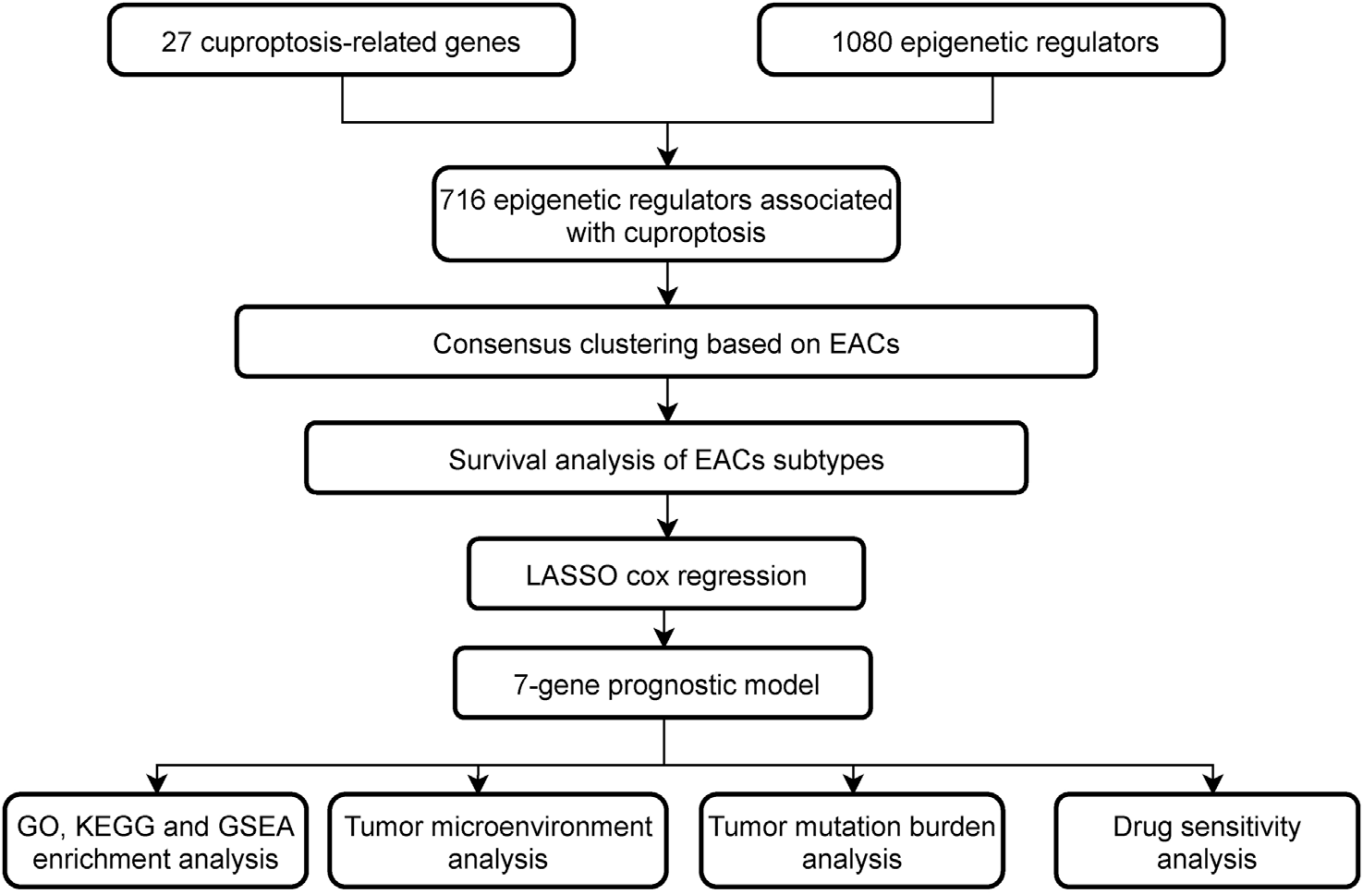
The study’s flow chart.

The study is summarized in a flowchart (Figure 1). Download and compile expression and clinical data: The RNA coding sequence data of 524 patients (41 normal and 483 tumor samples) were collected through the open-access TCGA-COAD database. Cuproptosis-related genes (CRGs) were sourced from the FerrDb database (FerrDb; http://www.zhounan.org/ferrdb/current/), while epigenetic regulators (EpiRegs) were discovered by exploring the GO term “histone modification” (https://www.yeastgenome.org/go/GO:0016570), according to a previous study (Yang et al., 2015), and the EpiFactors database (Supplementary Table S1) (Medvedeva et al., 2015). In order to identify epigenetic regulators associated with cuproptosis (EACs), the association between the epigenetic regulators and genes relevant to cuproptosis was evaluated using Pearson correlation. The data were analysed using R software (R Software vers. 4.2.2, The R Foundation for Statistical Computing, Vienna, Austria).

### Identifying EACs clusters with consensus clustering

The consensus clustering algorithm was employed using the ConsensusClusterPlus R tool to determine the number of clusters and their stability (Wilkerson and Hayes, 2010). In addition, we used the “survminer” program to do KM survival analysis on various patient clusters with EACs.

### Developing a prognostic model using EACs expression

To build a prognostic model, we randomly split the 455 COAD samples into a training set (*n* = 228) and a testing set (*n* = 227). Using the glmnet package in R (Xu et al., 2012), we performed the Univariate Cox regression and LASSO regression analysis on the EACs in the training set and identified 7 genes that contributed to the final prognostic model. Based on the expression levels and regression coefficients of these genes, we computed the risk score for each COAD sample. We classified the samples into high-risk and low-risk groups based on the median risk score. We assessed the survival differences between the two groups of samples in the training set, testing set and the whole dataset using Kaplan-Meier curves and log-rank tests. We also plotted a correlation heatmap of EACs and cuproptosis related genes using the pheatmap package in R. Furthermore, we evaluated the impacts of risk score and other clinical characteristics on the prognosis of COAD patients using univariate and multivariate Cox regression analysis. We estimated the AUC (area under the curve) of the prognostic model at different time points using the survivalROC package in R, and contrasted it with other clinical characteristics. We also computed the C-index (concordance index) of the prognostic model to assess its predictive ability. Finally, we built a prognostic prediction model based on risk score and other clinical characteristics using the rms package in R, and plotted a corresponding nomogram (Park, 2018).

### Enrichment functional analysis

We used the R package limma to perform differential analysis on the gene expression data of high-risk group and low-risk group, setting the screening criteria as |log2 fold change| > 1 and *p*-value <0.05, to identify the differentially expressed genes (DEGs). Then, we used the R package clusterProfiler to perform functional enrichment analysis on DEGs, including GO (Gene Ontology), KEGG (Kyoto Encyclopedia of Genes and Genomes) pathway and GSEA (Gene Set Enrichment Analysis) analysis, setting *p*-value <0.05 as significant enrichment.

### Relationship between risk score and tumor microenvironment

To explore the relationship between risk score and tumor microenvironment (TME), we used the ESTIMATE algorithm to calculate the immuneScore, stromalScore and ESTIMATEScore of each sample, reflecting the abundance of stromal cells and immune cells in the tumor microenvironment. We also used the CIBERSORT algorithm to estimate the relative proportions of 22 immune cell subgroups in each sample, and compared the immune cell subgroups differences between the two groups. Immune function was examined using single-sample gene set enrichment analysis (ssGSEA), to determine if there were any variations between groups. The Tumor Immune Dysfunction and Exclusion (TIDE, http://tide.dfci.harvard.edu/) is a computational tool that can predict the response to immune checkpoint blockade therapy from gen e expression data. Finally, we used the TIDE algorithm to calculate the TIDE score of each sample, reflecting the degree of tumor immune escape. *p*-values less than 0.05 were considered statistically significant.

### Analyzing the tumor mutation burden

We calculated the TMB values for each patient using the maftools package in R, and divided the patients into high-TMB group and low-TMB group. Subsequently, we compared the gene mutation profiles of the two groups. In addition, we compared the overall Survival rates of the two groups using Kaplan-Meier curves.

### Drug sensitivity analysis of high-risk and low-risk groups

To assess the differences in chemotherapeutic drug sensitivity between the high-risk and low-risk groups, we analyzed the data using the oncoPredict R package (Maeser et al., 2021). Then, we computed the mean IC50 values of each chemotherapeutic drug for both groups. We applied The Wilcoxon Rank Sum Test to compare the IC50 values of each drug across the two groups, and set the significance level at 0.05.

## Results

### Identification of two molecular subtypes of EACs in COAD

**FIGURE 2:**
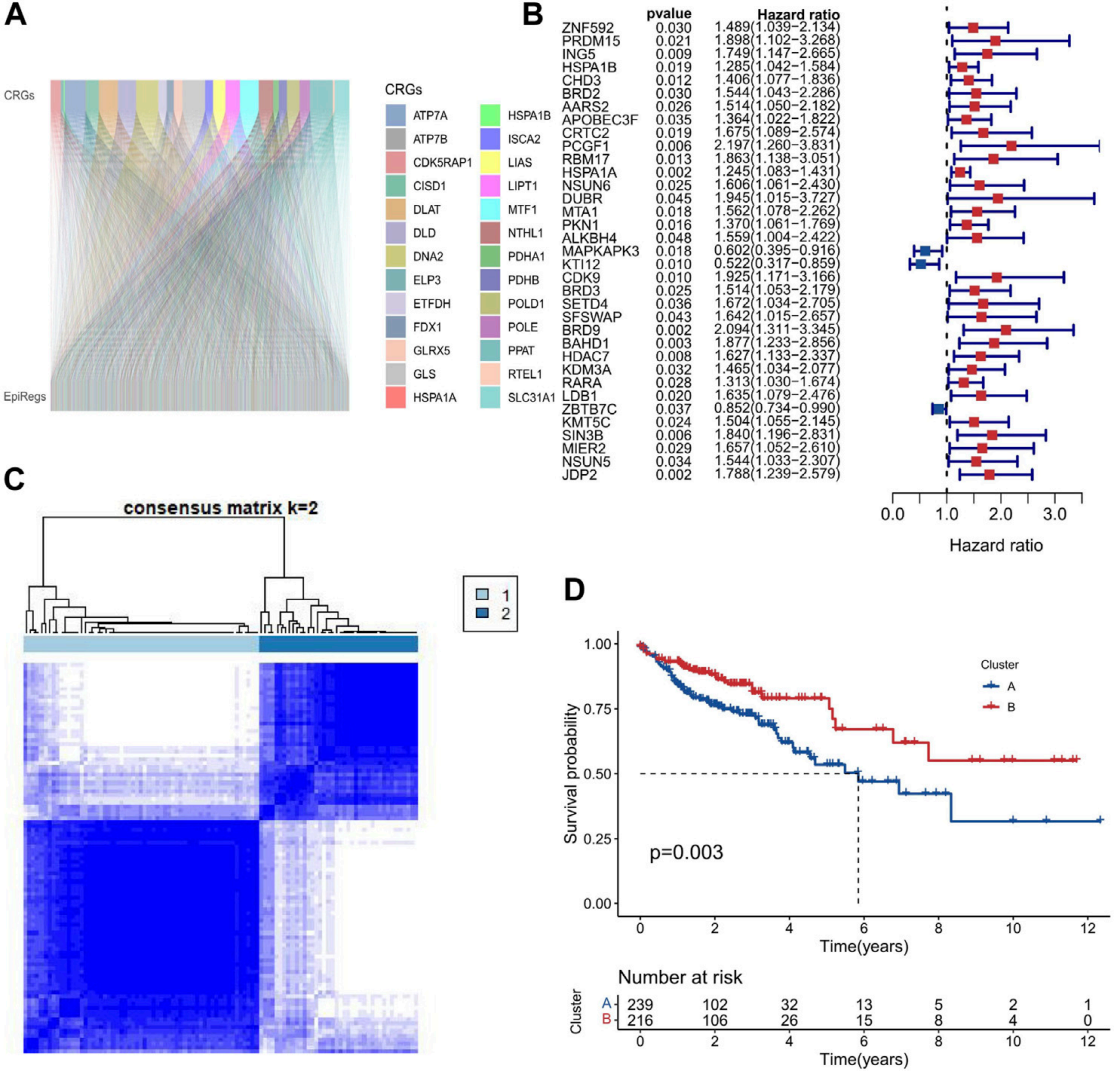
Two molecular subtypes of EACs in COAD. **(A)** We calculated the Pearson correlation coefficients between 27 genes involved in cuproptosis and 716 epigenetic regulators in COAD samples. The Sankey diagram shows that cuproptosis is strongly correlated with epigenetic regulation in COAD. **(B)** We performed Univariate Cox regression analysis to identify 35 EACs that are associated with survival in COAD samples. The forest plot shows the hazard ratios and 95% confidence intervals of these EACs. The red and blue dots represent the patients with HR values greater than 1 and less than 1, respectively. A higher HR value indicates a worse prognosis. **(C)** We used the ConsensusClusterPlus tool to cluster COAD samples based on the expression patterns of 35 EACs. The optimal number of clusters was k = 2. **(D)** The Kaplan-Meier survival curves for COAD samples with A and B subtypes. A subtype had a significantly worse survival than the B subtype.

In order to determine the epigenetic regulators linked to cuproptosis, we performed a Pearson correlation analysis between 27 cuproptosis-related genes and 1080 epigenetic regulators in COAD samples (*p* ≤ 0.001 and Pearson correlation coefficient ≥0.4). As shown in Figure 2A, we found 716 epigenetic regulators that were closely correlated with cuproptosis (EACs), indicating a strong association between cuproptosis and epigenetic regulators in COAD. Subsequently, Univariate Cox regression analysis was used to identify 35 genes that are connected to survival (Figure 2B). Using the R software tool “ConsensusClusterPlus,” 458 COAD samples were clustered into two groups based on the expression patterns of the 35 prognostic-related EACs. Since the classification was accurate and stable at k = 2, the samples were split into A and B subtypes (Figure 2C). The survival times of samples with the A subtype were lower than those of samples with the B subtype (*p* = 0.003; Figure 2D). The division into subtypes based on EACs could provide valuable prognostic information and guide personalized treatment strategies in COAD.

### Developing a prognostic model using EACs expression

**FIGURE 3:**
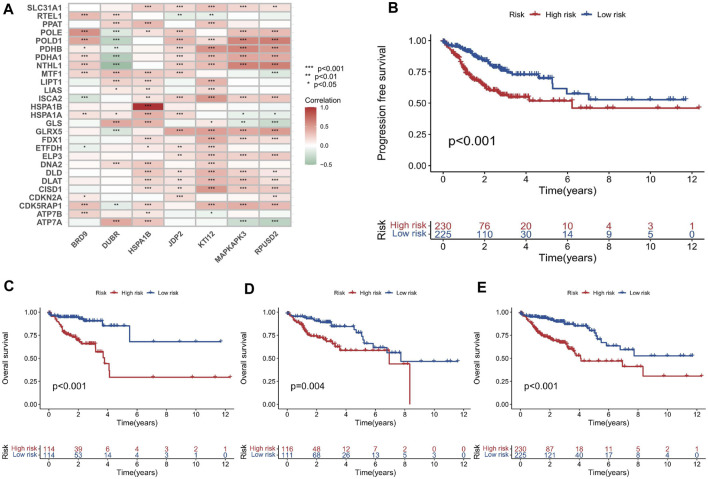
Development and evaluation of the prognostic model based on the EACs. **(A)** Heatmap illustrating the correlation between the 7 EACs and cuproptosis-related genes in COAD samples. **(B–E)** Kaplan-Meier survival plots for overall survival and progression-free survival in the training set, testing set, and entire set.

**FIGURE 4:**
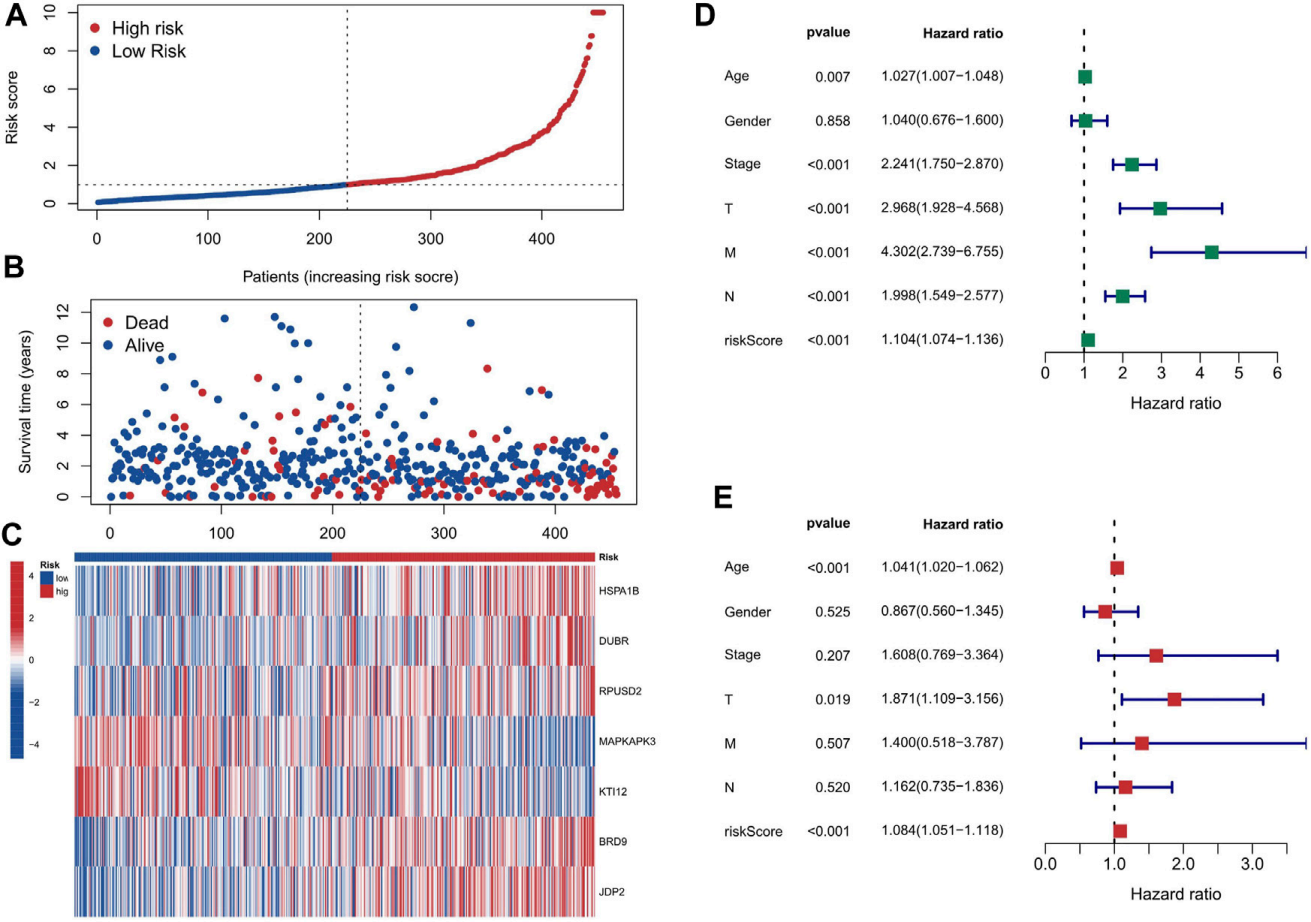
The impact of risk score based on EACs on the prognosis of COAD patients. **(A, B)** The distribution of each COAD patient’s risk score and survival status. **(C)** The expression levels of 5 genes are upregulated, while those of KTI12 and MAPKAPK3 are downregulated, as the risk score increases. **(D)** The Univariate Cox Regression Analysis indicates that risk score is one of the most important factors affecting the prognosis of COAD patients. **(E)** The Multivariate Cox Regression Analysis confirms that risk score is one of the most important factors affecting the prognosis of COAD patients.

**FIGURE 5:**
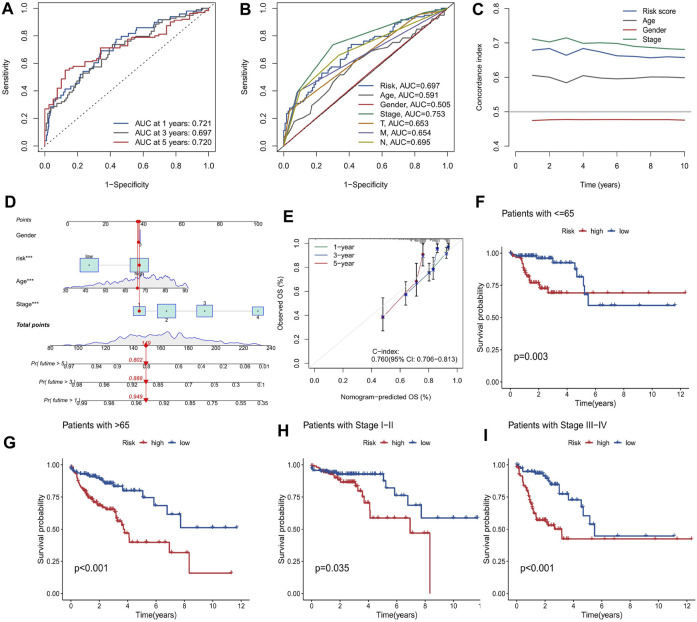
Evaluation of the prognostic model for COAD patients. **(A)** The performance of the prognostic model in predicting 1-, 3-, and 5-year OS, measured by the AUC values. **(B)** The comparison of the prognostic model and clinical variables in predicting 3-year OS, measured by the AUC values. **(C)** The agreement between the observed and predicted probabilities OS, measured by the calibration curve. **(D)** The nomogram that integrates the prognostic model and clinical variables to estimate the survival probability of COAD patients at 1, 3, and 5 years **(E)** The agreement between the observed and predicted probabilities of 3-year OS, measured by the calibration curve. **(F–I)** The survival analysis of the low and high risk groups in different subgroups of COAD patients, measured by the Kaplan-Meier curves: **(F)** Age<65 years, **(G)** Age≥65 years, **(H)** stage Ⅰ-Ⅱ, and **(I)** stage Ⅲ-Ⅳ.

First, the 455 COAD samples were split into a training group (n = 228) and a testing cohort (*n* = 227) using randomization. The training and test sets had the similar clinical features (Supplementary Table S2). Under the Univariate Cox regression and LASSO regression analysis, 7 genes were incorporated into the model. Each COAD sample was assigned a risk score based on the following formula: risk score = (0.3923) * HSPA1B+ (1.2941) * DUBR + (1.1061) * RPUSD2 + (−1.4366) * MAPKAPK3 + (−0.8613) * KTI12 + (0.6606) * BRD9 + (0.7803) * JDP2. The samples were separated into two groups based on their median risk score: high-risk and low-risk. The correlation between the 7 genes and cuproptosis related genes is shown in a heatmap (Figure 3A). Interestingly, among the 7 genes, HSPA1B is both a cuproptosis related gene and an epigenetic regulator. HSPA1B is a member of the HSP70 family. Increased HSPA1B expression and decreased HSPA1B promoter methylation level are both associated with a poor prognosis in colorectal cancer (Guan et al., 2021). Survival analysis using Kaplan-Meier curves revealed that the low-risk group had significantly better survival outcomes than the high-risk group in the training set (*p* < 0.001), testing set (*p* = 0.004), and the low-risk group also had significantly better outcomes than the high-risk group for the entire set in the progression-free survival analysis (*p* < 0.001) (Figures 3B–E). The significant differences in survival outcomes and progression-free survival between the low-risk and high-risk categories imply that risk grouping based on the EACs may help in the prediction of COAD patients’ prognosis. As the risk score increased, the survival rate of the individuals decreased (Figures 4A,B). The expression levels of five genes were positively correlated with the risk score, while KTI12 and MAPKAPK3 showed a negative correlation (Figure 4C). In the investigations of risk score and clinicopathological characteristics, the Univariate and Multivariate Cox Regression Analyses yielded *p*-values of less than 0.001. These analyses determined that the risk score was one of the most significant prognostic factors (Figures 4D,E). The prognostic model’s AUC values for predicting 1-, 3-, and 5-year OS were 0.721, 0.697, and 0.720, respectively (Figure 5A). The 3-year AUC of the prognostic model was 0.697, which was significantly higher than that of age (AUC = 0.591), gender (AUC = 0.505), T stage (AUC = 0.653), M stage (AUC = 0.654), N stage (AUC = 0.695) (Figure 5B). The 1-year C-index of 0.68 showed that the prognostic model performed well (Figure 5C). Additionally, the nomogram was created to forecast the survival rate of COAD patients (1, 3, and 5 years) based on fundamental clinical characteristics and risk score (Figure 5D). The nomogram exhibited a C-index of 0.760 (Figure 5E), indicating its good predictive ability for the survival rate of COAD patients at 1, 3, and 5 years. Next, we performed OS survival analysis for two risk groups in COAD patient subgroups. We found that the risk score was a potential prognostic indicator in COAD patients in all subgroups: Age<65 years (*p* = 0.003), Age≥65 years (*p* < 0.001), stage Ⅰ-Ⅱ (*p* = 0.035), and stage Ⅲ-Ⅳ (*p* < 0.001). Our results also demonstrated better predictive power in stage Ⅲ-Ⅳ than in stage Ⅰ-Ⅱ (Figures 5F–I).

### Enrichment functional analysis of the DEGs between high and low risk groups

**FIGURE 6:**
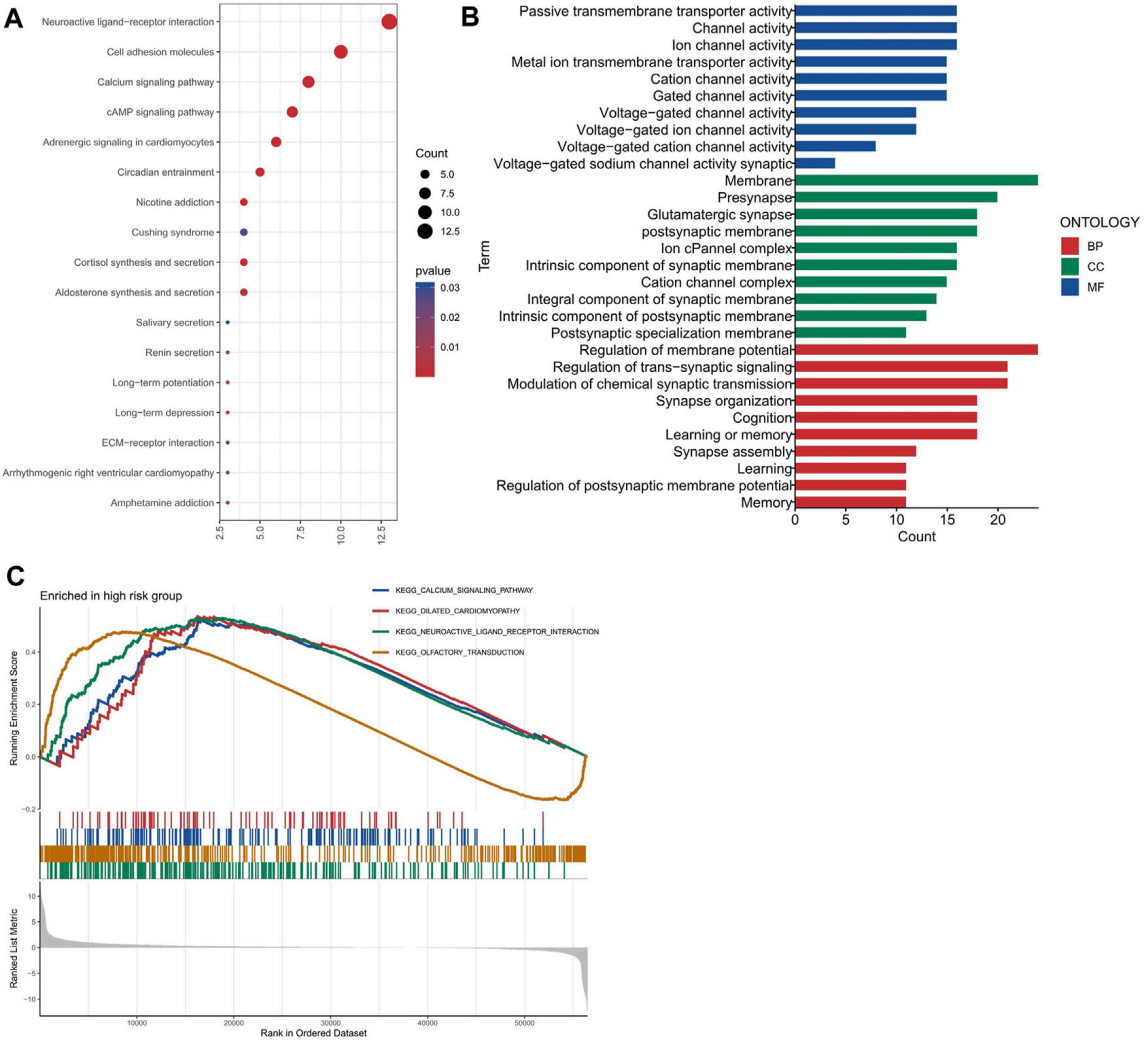
Functional enrichment analysis of the differentially expressed genes between the high and low risk groups. **(A)** The most significant KEGG pathways enriched by the differentially expressed genes. **(B)** The most significant GO terms enriched by the differentially expressed genes for each category (BP, CC, and MF). **(C)** The GSEA enrichment plots for four pathways.

To investigate the biological roles and pathways associated with the risk score, we identified 246 DEGs (244 genes with increased expression and 2 genes with decreased expression) between the groups with high and low risk. We conducted enrichment analyses using GO, KEGG, and GSEA methods. The DEGs were significantly associated with some KEGG pathways, such as Neuroactive ligand-receptor interaction, Cell adhesion molecules, Calcium signaling pathway and cAMP signaling pathway, etc., as shown in Figure 6A. Neuroactive ligands and their corresponding receptors can exert a pivotal influence on cellular signaling cascades, regulating cuproptosis and other forms of cellular demise. Cell adhesion molecules play a crucial role in cellular adhesion and interactions (Janiszewska et al., 2020). Perturbations in their expression can disrupt intercellular adhesion capabilities, subsequently affecting cellular proliferation, survival, and apoptotic processes (Harjunpaa et al., 2019), including cuproptosis, within the landscape of colon adenocarcinoma. Furthermore, aberrant calcium signaling and cAMP signaling pathways appear intricately involved in the delicate balance between cell proliferation and apoptosis (Chin et al., 2002; Wang et al., 2019). Their dysregulation can contribute to cuproptosis and other cellular death mechanisms that underlie the development and progression of colon adenocarcinoma. Figure 6B displayed the significantly enriched GO items in the DEGs, including regulation of membrane potential, modulation of chemical synaptic transmission and regulation of trans-synaptic signaling for biological process (BP), and synaptic membrane, presynapse, postsynaptic membrane and glutamatergic synapse for cellular component (CC), and ion channel activity, channel activity and passive transmembrane transporter activity for molecular function (MF). The GSEA enrichment analysis strongly implicated four primary biological processes: neuroactive ligand-receptor interaction, olfactory transduction, calcium signaling pathway and dilated cardiomyopathy (Figure 6C).

### Relationship between risk score and tumor microenvironment

**FIGURE 7:**
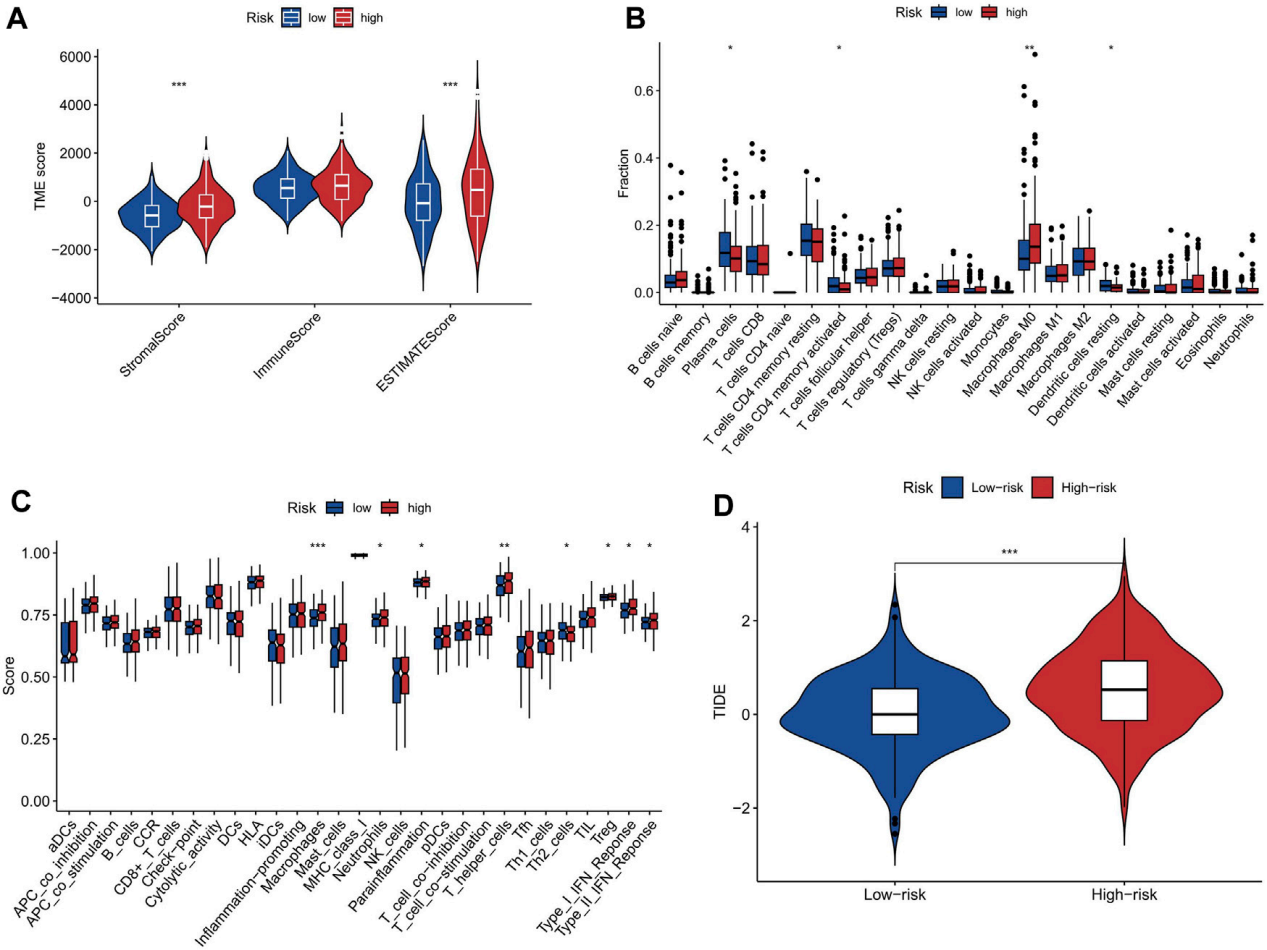
The correlation between risk score and tumor microenvironment. **(A)** ImmuneScore, StromalScore, and ESTIMATEScore in the two risk groups. **(B)** Abundance of immune cells in high-risk and low-risk groups. The abundance of M0 macrophages was significantly higher in the high-risk group than in the low-risk group. **(C)** Immune function scores in high-risk and low-risk groups. Macrophages score was significantly higher in the high-risk group than in the low-risk group. **(D)** Boxplots showing the distribution of TIDE score in the high-risk and low-risk groups. * represent *p* < 0.05; * * represent *p* < 0.01; * * *represent *p* < 0.001.

Stromal, tumor, and immune infiltrating cells make up the tumor microenvironment. The high-risk group exhibited significantly elevated levels of stromalScore and ESTIMATEScore in the TME, compared to the low-risk group, as shown in Figure 7A. The elevated stromalScore and ESTIMATEScore in the high-risk group potentially reflect the activation of various biological processes within the TME, including angiogenesis, extracellular matrix remodeling, and immune evasion mechanisms (Anderson and Simon, 2020). These processes contribute to tumor progression and may serve as potential targets for therapeutic interventions. In the high-risk group, the abundance of M0 macrophages was comparatively higher when compared to the low-risk group (Figure 7B), and the immune function analysis also showed that the high-risk group had higher macrophages score than the low-risk group (Figure 7C). The higher abundance of M0 macrophages in the high-risk group suggests a dysregulated immune response, as M0 macrophages are an intermediate stage in macrophage activation. It indicates that the immune system in high-risk individuals may be skewed towards an inflammatory state, which can potentially contribute to the development and progression of various diseases including cancer (Christofides et al., 2022). The analysis of our study demonstrates that there is a significant difference in TIDE scores between the high-risk and low-risk groups, with the high-risk group exhibiting significantly higher scores (Figure 7D). This finding suggests that the high-risk group is more prone to immune escape in the context of tumor-immune cell interactions. Understanding the underlying mechanisms of immune escape can guide the development of targeted therapies to overcome or prevent immune resistance.

### Analyzing the tumor mutation burden

**FIGURE 8:**
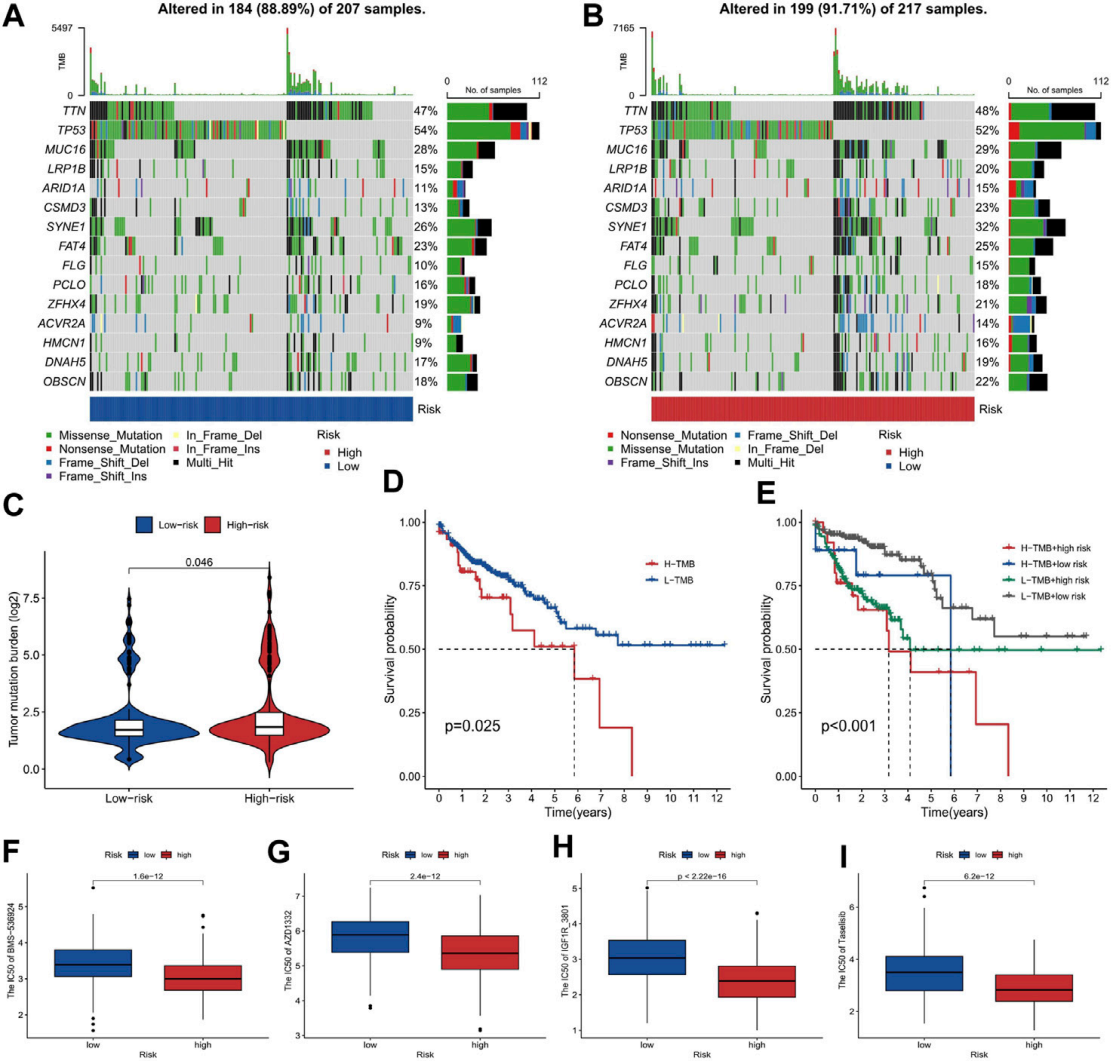
The tumor mutation burden and drug sensitivity for risk stratification in COAD. **(A, B)** The gene mutation frequency of the high-risk and low-risk groups. **(C)** The comparison of TMB between the high-risk and low-risk groups. **(D, E)** The survival probability of patients classified by TMB and risk score, shown as a Kaplan-Meier curve. **(F–I)** The sensitivity of high-risk and low-risk groups to chemotherapeutic drugs Taselisib, IGF1R_3801, AZD1332, and BMS-536924, shown as boxplots of IC50 values.

Our study on COAD using tumor mutation burden (TMB) analysis revealed a slightly higher TMB in the high-risk group compared to the low-risk group, particularly in the CSMD3 gene (Figures 8A–C). Firstly, our risk stratification based on TMB analysis demonstrates the accuracy of our model in categorizing patients into high- and low-risk groups. This has great potential for personalized treatment decisions in COAD patients. Additionally, the significantly higher mutation rate of CSMD3 in the high-risk group suggests its value as a prognostic biomarker for this disease. Further investigation into CSMD3 mutations could provide insights into underlying mechanisms associated with high-risk phenotypes. The analysis of our research reveals that patients with a high TMB combined with a high-risk score exhibit a lower survival probability compared to other patients (Figures 8D,E). By identifying patients with low TMB and high risk, we can potentially identify a subgroup with particularly poor prognosis. This information can aid clinicians in making more informed treatment decisions.

### Drug sensitivity analysis of high and low risk groups

The analysis of chemotherapeutic drug sensitivity between the high-risk and low-risk groups, as depicted in Figures 8F–I, has revealed significant differences in IC50 values. The patients with a higher risk exhibited lower IC50 values, indicating greater sensitivity to chemotherapeutic drugs such as Taselisib, IGF1R_3801, AZD1332, and BMS-536924 (Figures 8F–I). Our study provides evidence that high-risk patients, identified based on our model, are more responsive to these specific chemotherapeutic agents. These findings offer the potential to enhance treatment efficacy and optimize therapeutic outcomes in COAD cases.

## Discussion

The high incidence and death rate of COAD significantly necessitate the development of novel therapeutic methods for it. Identifying the vulnerability of COAD cells gives us more chances to find effective way to treat this tumor. All these rely on the progress in understanding the basic mechanism for COAD cells to live, proliferate, metastasize, and even die. To make the COAD cells die is the most intuitive way to cure the patients. The question is how to achieve it. Since the discovery that apoptosis is a type of regulated cell death, the cell death field is always a hotspot in biology and medicine (Galluzzi et al., 2018). Besides apoptosis, there have been many more modes of regulated cell deaths discovered in recent decades, such as ferroptosis (Liu and Gu, 2022b), necroptosis (Pasparakis and Vandenabeele, 2015), pyroptosis (Yu et al., 2021), and the very recently named cuproptosis (Tsvetkov et al., 2022). These cell death modalities have opened new windows to treat COAD. For example, inducing ferroptosis may be an efficient way to eliminate COAD cells (Yan et al., 2023). Necroptosis has been demonstrated to involve COAD progression (Han et al., 2018). The role of cuproptosis in COAD and the efficacy of targeting it to treat COAD is largely elusive. However, there are at least several theoretical rationales making cuproptosis an attractive biological process targeting which may greatly benefit the COAD patients. First, cuproptosis represents a novel cell death pathway caused by copper and the lipoylation of TCA cycle proteins. This mechanism of action is totally different from other regulated cell deaths, which exposes a unique vulnerability of tumor cells. Second, this distinct mechanism of cuproptosis makes it possible to combine cuproptosis-based therapy with other treatment methods, like chemotherapy and immunotherapy, in COAD therapy. Third, colon is an organ with active copper metabolism. Particularly, gut microbiota has been demonstrated to be involved in the uptake and metabolism of copper (Mu and Zhu, 2019; Pajarillo et al., 2021). Indeed, there is evidence that deregulated copper metabolism promotes colon tumorigenesis (Liao et al., 2020). Fourth, COAD is accompanied by dramatically changed cellular metabolism, including the TCA cycle in mitochondria (La Vecchia and Sebastian, 2020; Sedlak et al., 2023). Combination of the third point and fourth points reveals a fact that COAD cells may be more susceptible to cuproptosis and induction of cuproptosis may be a promising way to treat COAD. These speculations await further studies. To advance this field and boost the development of cuproptosis-based therapeutics in COAD, more knowledge must be obtained about the mechanism and regulation of cuproptosis. In addition, identification of more cuproptosis-associated genes and figuring out the pathological relevance of them will not only benefit the cuproptosis research, but also contribute to the translation of knowledge about cuproptosis into clinical usage (Liu et al., 2022a). In this study, we focused on COAD samples to identify cuproptosis-associated genes and investigated their prognostic value.

Epigenetics stands in the middle of the stage in biological research. In the recent 3 decades, epigenetics has greatly broadened our horizon about gene expression regulation. To date, epigenetic regulation of gene expression has been intensively studied. A number of epigenetic regulators are proved to be associated with almost all the physiological or pathological processes. Abundant drugs targeting epigenetic regulators and pathways have been developed to treat diverse disorders, including cancer (Campbell and Tummino, 2014; Ganesan et al., 2019). Particularly, the initiation and development of COAD are associated with various epigenetic alterations (Lao and Grady, 2011). However, the role of epigenetic regulators in cuproptosis has not been elucidated. To leverage the exciting potential of targeting epigenetic regulators and cuproptosis in treating COAD, in this study we focused on linking epigenetic regulators, cuproptosis, and COAD together.

By performing co-expression analysis, we found that in COAD patients, a lot of epigenetic regulators are correlated with prognostic prediction model. Furthermore, the Univariate Cox regression and LASSO regression analysis on those genes revealed a gene set composed of 7 epigenetic regulators BRD9, DUBR, HSPA1B, JDP2, KTI12, MAPKAPK3, and RPUSD2, which may establish a risk score model with prognostic value in these patients. The followed validation demonstrated the high efficiency of this epigenetic signature in predicting the survival of COAD patients. This model performed better in patients with stage III-IV than in those with stage I-II. This may be due to that this set of genes mainly functions in the metastasis stage but not the primary stage of this cancer. Interestingly, the prognostic value of this signature is much higher in patients older than 65. This is consistent with a novel idea that ageing is caused by the alteration of epigenetic state of the cell (Dongxin Zhao and Chen, 2022; Yang et al., 2023). Whether cuproptosis participates in the regulation of metastasis of COAD and the ageing process is unknown. However, these are two interesting directions for the future cuproptosis study.

To investigate the potential function of this gene set, we unexpectedly found that it is associated with the tumor microenvironment, particularly the immune cell infiltration. This result is understandable from the epigenetic factor angle, as epigenetics is critical for modulating cancer immunity (Cao and Yan, 2020). About the relationship between cuproptosis and tumor immunity, we can get some clues from other regulated cell death modes. For example, necroptosis of tumor cell can induce anti-tumor immunity (Meng et al., 2016). Pyroptosis is a host immune defense against pathogen infection. It is also related to tumor immunity (Li et al., 2021). Recently, more and more data showed that ferroptosis also participates in the mediation of tumor immunity (Gong et al., 2022). Based on these facts, it is reasonable to hypothesize that cuproptosis has a role in regulating tumor immunity in COAD. Additionally, we revealed that this signature is correlated with the tumor mutation profile and burden of these patients. It is possible that these mutated genes are contributors or regulators of cuproptosis. A possibility is that COAD cells have different sensitivity to cuproptosis compared to normal cells partly due to the gene mutation profile and the genome instability.

About the basic functions of the 7 genes, some of them have been studied a lot. BRD9 is a bromodomain containing chromatin modifying protein. It is proved to play an important role in colon cancer (Kapoor et al., 2022; Zhu et al., 2023). Interestingly, a recent study by Zhu et al., 2023 found that BRD9 is a critical regulator of glycolysis in colon cancer. Glycolysis fuels the TCA cycle and cuproptosis is caused by the lipoylated TCA cycle proteins. Therefore, BRD9 may regulate cuproptosis by promoting glycolysis. DUBR is a long non-coding RNA involved in several cancer types (Liu et al., 2022b; Han et al., 2022; Nie et al., 2022). However, whether DUBR promotes or suppresses cancer progression is controversial in different contexts. Its role in COAD is largely unknown. HSPA1B is shared by the cuproptosis-related gene set and the epigenetic regulator set. How it is associated with these two distinct molecular processes needs more exploration. JDP2 is a transcription repressor and is repressed by UHRF1 in colon cancer (Hong et al., 2022). The acetylation-deficient mutant UHRF1 4 KR loses the ability to inhibit JDP2 and the latter gene will be upregulated to suppress colon cancer cell proliferation. Whether this tumor suppressive effect of JDP2 results from enhanced cuproptosis is still elusive. The rest three genes KTI12, MAPKAPK3, and RPUSD2 haven’t been well-studied in colon cancer. Taken together, further investigation is warranted to clarify the functions of these genes in colon cancer, cuproptosis, and epigenetics. A limitation of our study is the lack of direct experimental evidence for the connection between cuproptosis and epigenetic regulators in colon adenocarcinoma, and we design to conduct more experiments in the future to confirm our deductions.

To conclude, we developed an effective prognostic model in COAD, which consists of 7 cuproptosis-related epigenetic regulators. We wish that this model can not only benefit the treatment of COAD patients, but also advance the research in the cuproptosis field.

## Data Availability

The original contributions presented in the study are included in the article/Supplementary Material, further inquiries can be directed to the corresponding authors.

## References

[B1] Anderson N. M. Simon M. C. (2020). The tumor microenvironment. Curr. Biol. 30 (16), R921–R925. 10.1016/j.cub.2020.06.081 32810447 PMC8194051

[B2] Campbell R. M. Tummino P. J. (2014). Cancer epigenetics drug discovery and development: the challenge of hitting the mark. J. Clin. Investigation 124 (1), 64–69. 10.1172/JCI71605 PMC3871251 24382391

[B3] Cao J. Yan Q. (2020). Cancer epigenetics, tumor immunity, and immunotherapy. Trends Cancer 6 (7), 580–592. 10.1016/j.trecan.2020.02.003 32610068 PMC7330177

[B4] Cao S. H. Wang Q. Sun Z. Z. Zhang Y. Liu Q. Q. Huang Q. (2023). Role of cuproptosis in understanding diseases. Hum. Cell. 36, 1244–1252. 10.1007/s13577-023-00914-6 37154876 PMC10165592

[B5] Cavalli G. Heard E. (2019). Advances in epigenetics link genetics to the environment and disease. Nature 571 (7766), 489–499. 10.1038/s41586-019-1411-0 31341302

[B6] Chin K. V. Yang W. L. Ravatn R. Kita T. Reitman E. Vettori D. (2002). Reinventing the wheel of cyclic AMP - novel mechanisms of cAMP signaling. Ann. N. Y. Acad. Sci. 968, 49–64. 10.1111/j.1749-6632.2002.tb04326.x 12119267

[B7] Christofides A. Strauss L. Yeo A. Cao C. Charest A. Boussiotis V. A. (2022). The complex role of tumor-infiltrating macrophages. Nat. Immunol. 23 (8), 1148–1156. 10.1038/s41590-022-01267-2 35879449 PMC10754321

[B8] Dongxin Zhao S. C. Chen S. (2022). Failures at every level: breakdown of the epigenetic machinery of aging. Life Med. 1 (2), 81–83. 10.1093/lifemedi/lnac016 PMC11749257 39871931

[B9] Galluzzi L. Vitale I. Aaronson S. A. Abrams J. M. Adam D. Agostinis P. (2018). Molecular mechanisms of cell death: recommendations of the nomenclature committee on cell death 2018. Cell. death Differ. 25 (3), 486–541. 10.1038/s41418-017-0012-4 29362479 PMC5864239

[B10] Ganesan A. Arimondo P. B. Rots M. G. Jeronimo C. Berdasco M. (2019). The timeline of epigenetic drug discovery: from reality to dreams. Clin. Epigenetics 11 (1), 174. 10.1186/s13148-019-0776-0 31791394 PMC6888921

[B11] Gao Z. Y. Zhou L. K. Hua S. Y. Wu H. Luo L. Z. Li L. B. (2020). miR-24-3p promotes colon cancer progression by targeting ING1. Signal Transduct. Tar 5 (1), 171. 10.1038/s41392-020-0206-y PMC7447644 32843621

[B12] Gong C. D. Ji Q. K. Wu M. J. Tu Z. W. Lei K. J. Luo M. (2022). Ferroptosis in tumor immunity and therapy. J. Cell. Mol. Med. 26 (22), 5565–5579. 10.1111/jcmm.17529 36317423 PMC9667519

[B13] Guan Y. F. Zhu X. J. Liang J. J. Wei M. Huang S. Pan X. F. (2021). Upregulation of hspa1a/HSPA1B/HSPA7 and downregulation of HSPA9 were related to poor survival in colon cancer. Front. Oncol. 11, 749673. 10.3389/fonc.2021.749673 34765552 PMC8576338

[B14] Han Q. R. Ma Y. Wang H. Dai Y. Chen C. H. Liu Y. W. (2018). Resibufogenin suppresses colorectal cancer growth and metastasis through RIP3-mediated necroptosis. J. Transl. Med. 16, 201. 10.1186/s12967-018-1580-x 30029665 PMC6053767

[B15] Han Z. H. Li D. Yang Y. Zhang H. (2022). LINC-DUBR suppresses malignant progression of ovarian cancer by downregulating miR-107 to induce SMAC expression. J. Healthc. Eng. 2022, 4535655. 10.1155/2022/4535655 35281523 PMC8913066

[B16] Harjunpaa H. Asens M. L. Guenther C. Fagerholm S. C. (2019). Cell adhesion molecules and their roles and regulation in the immune and tumor microenvironment. Front. Immunol. 10, 1078. 10.3389/fimmu.2019.01078 31231358 PMC6558418

[B17] Hong Y. J. Park J. Hahm J. Y. Kim S. H. Lee D. H. Park K. S. (2022). Regulation of UHRF1 acetylation by TIP60 is important for colon cancer cell proliferation. Genes. Genom 44 (11), 1353–1361. 10.1007/s13258-022-01298-x PMC9569301 35951156

[B18] Janiszewska M. Primi M. C. Izard T. (2020). Cell adhesion in cancer: beyond the migration of single cells. J. Biol. Chem. 295 (8), 2495–2505. 10.1074/jbc.REV119.007759 31937589 PMC7039572

[B19] Kapoor S. Damiani E. Wang S. Dharmanand R. Tripathi C. Perez J. E. T. 2022, BRD9 inhibition by natural polyphenols targets DNA damage/repair and apoptosis in human colon cancer cells. Nutrients 2022, 14, 4317, 10.3390/nu14204317 20). PMC9610492 36297001

[B20] Koch A. Joosten S. C. Feng Z. de Ruijter T. C. Draht M. X. Melotte V. (2018). Author correction: analysis of DNA methylation in cancer: location revisited. Nat. Rev. Clin. Oncol. 15 (7), 467. 10.1038/s41571-018-0028-9 29713045

[B21] La Vecchia S. Sebastian C. (2020). Metabolic pathways regulating colorectal cancer initiation and progression. Seminars Cell. & Dev. Biol. 98, 63–70. 10.1016/j.semcdb.2019.05.018 31129171

[B22] Lao V. V. Grady W. M. (2011). Epigenetics and colorectal cancer. Nat. Rev. Gastro Hepat. 8 (12), 686–700. 10.1038/nrgastro.2011.173 PMC3391545 22009203

[B23] Li L. S. Jiang M. X. Qi L. Wu Y. M. Song D. F. Gan J. Q. (2021). Pyroptosis, a new bridge to tumor immunity. Cancer Sci. 112 (10), 3979–3994. 10.1111/cas.15059 34252266 PMC8486185

[B24] Liao Y. Zhao J. J. Bulek K. Tang F. Q. Chen X. Cai G. (2020). Inflammation mobilizes copper metabolism to promote colon tumorigenesis via an IL-17-STEAP4-XIAP axis. Nat. Commun. 11 (1), 900. 10.1038/s41467-020-14698-y 32060280 PMC7021685

[B25] Liu Y. Gu W. (2022a). The complexity of p53-mediated metabolic regulation in tumor suppression. Seminars cancer Biol. 85, 4–32. 10.1016/j.semcancer.2021.03.010 PMC8473587 33785447

[B26] Liu Y. Q. Gu W. (2022b). p53 in ferroptosis regulation: the new weapon for the old guardian. Cell. death Differ. 29 (5), 895–910. 10.1038/s41418-022-00943-y 35087226 PMC9091200

[B27] Liu Y. Q. Liu R. Yang F. Cheng R. J. Chen X. R. Cui S. F. (2017). miR-19a promotes colorectal cancer proliferation and migration by targeting TIA1. Mol. Cancer 16, 53. 10.1186/s12943-017-0625-8 28257633 PMC5336638

[B28] Liu Y. Q. Chen X. R. Cheng R. J. Yang F. Yu M. C. Wang C. (2018). The Jun/miR-22/HuR regulatory axis contributes to tumourigenesis in colorectal cancer. Mol. Cancer 17, 11. 10.1186/s12943-017-0751-3 29351796 PMC5775639

[B29] Liu Y. Q. Tavana O. Gu W. (2019). p53 modifications: exquisite decorations of the powerful guardian. J. Mol. Cell. Biol. 11 (7), 564–577. 10.1093/jmcb/mjz060 31282934 PMC6736412

[B30] Liu Y. Q. Liu Y. Ye S. J. Feng H. J. Ma L. J. (2022a). Development and validation of cuproptosis-related gene signature in the prognostic prediction of liver cancer. Front. Oncol. 12, 985484. 10.3389/fonc.2022.985484 36033443 PMC9413147

[B31] Liu S. Bu X. Y. Kan A. Luo L. Xu Y. J. Chen H. L. (2022b). SP1-induced lncRNA DUBR promotes stemness and oxaliplatin resistance of hepatocellular carcinoma via E2F1-CIP2A feedback. Cancer Lett. 528, 16–30. 10.1016/j.canlet.2021.12.026 34958891

[B32] Maeser D. Gruener R. F. Huang R. S. (2021). oncoPredict: an R package for predicting *in vivo* or cancer patient drug response and biomarkers from cell line screening data. Brief. Bioinform 22 (6), bbab260. 10.1093/bib/bbab260 34260682 PMC8574972

[B33] Medvedeva Y. A. Lennartsson A. Ehsani R. Kulakovskiy I. V. Vorontsov I. E. Panahandeh P. (2015). EpiFactors: A comprehensive database of human epigenetic factors and complexes. Database-Oxford 2015, bav067. 10.1093/database/bav067 26153137 PMC4494013

[B34] Meng M. B. Wang H. H. Cui Y. L. Wu Z. Q. Shi Y. Y. Zaorsky N. G. (2016). Necroptosis in tumorigenesis, activation of anti-tumor immunity, and cancer therapy. Oncotarget 7 (35), 57391–57413. 10.18632/oncotarget.10548 27429198 PMC5302997

[B35] Michniewicz F. Saletta F. Rouaen J. R. C. Hewavisenti R. V. Mercatelli D. Cirillo G. (2021). Copper: an intracellular achilles' heel allowing the targeting of epigenetics, kinase pathways, and cell metabolism in cancer therapeutics. Chemmedchem 16 (15), 2315–2329. 10.1002/cmdc.202100172 33890721

[B36] Mu C. L. Zhu W. Y. (2019). Antibiotic effects on gut microbiota, metabolism, and beyond. Appl. Microbiol. Biot. 103 (23-24), 9277–9285. 10.1007/s00253-019-10165-x 31701196

[B37] Nie W. Hu M. J. Zhang Q. Lu J. Qian F. F. Zhang L. L. (2022). DUBR suppresses migration and invasion of human lung adenocarcinoma cells via ZBTB11-mediated inhibition of oxidative phosphorylation. Acta Pharmacol. Sin. 43 (1), 157–166. 10.1038/s41401-021-00624-5 33758355 PMC8724295

[B38] Pajarillo E. A. B. Lee E. Kang D. K. (2021). Trace metals and animal health: interplay of the gut microbiota with iron, manganese, zinc, and copper. Anim. Nutr. 7 (3), 750–761. 10.1016/j.aninu.2021.03.005 34466679 PMC8379138

[B39] Park S. Y. (2018). Nomogram: an analogue tool to deliver digital knowledge. J. Thorac. Cardiov Sur 155 (4), 1793. 10.1016/j.jtcvs.2017.12.107 29370910

[B40] Pasparakis M. Vandenabeele P. (2015). Necroptosis and its role in inflammation. Nature 517 (7534), 311–320. 10.1038/nature14191 25592536

[B41] Saldana-Meyer R. Recillas-Targa F. (2011). Transcriptional and epigenetic regulation of the p53 tumor suppressor gene. Epigenetics-Us 6 (9), 1068–1077. 10.4161/epi.6.9.16683 21814038

[B42] Sedlak J. C. Yilmaz O. H. Roper J. (2023). Metabolism and colorectal cancer. Annu. Rev. pathology 18, 467–492. 10.1146/annurev-pathmechdis-031521-041113 PMC9877174 36323004

[B43] Shanbhag V. C. Gudekar N. Jasmer K. Papageorgiou C. Singh K. Petris M. J. (2021). Copper metabolism as a unique vulnerability in cancer. Bba-Mol Cell. Res. 1868 (2). 10.1016/j.bbamcr.2020.118893 PMC7779655 33091507

[B44] Sharma S. Kelly T. K. Jones P. A. (2010). Epigenetics in cancer. Carcinogenesis 31 (1), 27–36. 10.1093/carcin/bgp220 19752007 PMC2802667

[B45] Siegel R. L. Wagle N. S. Cercek A. Smith R. A. Jemal A. (2023a). Colorectal cancer statistics, 2023. Ca-Cancer J. Clin. 73, 233–254. 10.3322/caac.21772 36856579

[B46] Siegel R. L. Miller K. D. Wagle N. S. Jemal A. (2023b). Cancer statistics, 2023. Ca-Cancer J. Clin. 73 (1), 17–48. 10.3322/caac.21763 36633525

[B47] Tsvetkov P. Coy S. Petrova B. Dreishpoon M. Verma A. Abdusamad M. (2022). Copper induces cell death by targeting lipoylated TCA cycle proteins. Science 375 (6586), 1254–1261. 10.1126/science.abf0529 35298263 PMC9273333

[B48] Wang W. Yu S. Y. Huang S. Deng R. Ding Y. S. Wu Y. Y. (2019). A complex role for calcium signaling in colorectal cancer development and progression. Mol. Cancer Res. 17 (11), 2145–2153. 10.1158/1541-7786.MCR-19-0429 31366605

[B49] Wilkerson M. D. Hayes D. N. (2010). ConsensusClusterPlus: A class discovery tool with confidence assessments and item tracking. Bioinformatics 26 (12), 1572–1573. 10.1093/bioinformatics/btq170 20427518 PMC2881355

[B50] Wu G. Y. Peng H. Tang M. L. Yang M. S. Z. Wang J. Hu Y. M. (2021). ZNF711 down-regulation promotes CISPLATIN resistance in epithelial ovarian cancer via interacting with JHDM2A and suppressing SLC31A1 expression. EBioMedicine 71. 10.1016/j.ebiom.2021.103558 PMC8441092 34521054

[B51] Xie J. M. Yang Y. N. Gao Y. B. He J. (2023). Cuproptosis: mechanisms and links with cancers. Mol. Cancer 22 (1), 46. 10.1186/s12943-023-01732-y 36882769 PMC9990368

[B52] Xiong C. Ling H. Hao Q. Cuproptosis Zhou X. (2023). p53-regulated metabolic cell death? Cell. death Differ. 30, 876–884. 10.1038/s41418-023-01125-0 36755067 PMC10070433

[B53] Xu C. J. van der Schaaf A. van't Veld A. A. Langendijk J. A. Schilstra C. (2012). Statistical validation of normal tissue complication probability models. Int. J. Radiat. Oncol. 84 (1), E123–E129. 10.1016/j.ijrobp.2012.02.022 22541961

[B54] Xu J. H. Hu Z. A. Cao H. Zhang H. Luo P. Zhang J. (2022). Multi-omics pan-cancer study of cuproptosis core gene FDX1 and its role in kidney renal clear cell carcinoma. Front. Immunol. 13, 981764. 10.3389/fimmu.2022.981764 36605188 PMC9810262

[B55] Yan H. Talty R. Aladelokun O. Bosenberg M. Johnson C. H. (2023). Ferroptosis in colorectal cancer: A future target? Br. J. cancer 128 (8), 1439–1451. 10.1038/s41416-023-02149-6 36703079 PMC10070248

[B56] Yang Z. Jones A. Widschwendter M. Teschendorff A. E. (2015). An integrative pan-cancer-wide analysis of epigenetic enzymes reveals universal patterns of epigenomic deregulation in cancer. Genome Biol. 16, 140. 10.1186/s13059-015-0699-9 26169266 PMC4501092

[B57] Yang J. H. Hayano M. Griffin P. T. Amorim J. A. Bonkowski M. S. Apostolides J. K. (2023). Loss of epigenetic information as a cause of mammalian aging. Cell. 186 (2), 305–326.e27. 10.1016/j.cell.2022.12.027 36638792 PMC10166133

[B58] Yu P. Zhang X. Liu N. Tang L. Peng C. Chen X. (2021). Pyroptosis: mechanisms and diseases. Signal Transduct. Tar 6 (1), 128. 10.1038/s41392-021-00507-5 PMC8005494 33776057

[B59] Zhu Q. S. Gu X. Wei W. Wu Z. Gong F. Q. Dong X. Q. (2023). BRD9 is an essential regulator of glycolysis that creates an epigenetic vulnerability in colon adenocarcinoma. Cancer Med-Us 12 (2), 1572–1587. 10.1002/cam4.4954 PMC9883419 35778964

